# Supplementary material for: The causal relationship between the human gut microbiota and pyogenic arthritis: a Mendelian randomization study
Source: Front Cell Infect Microbiol. 2024 Nov 26;14:1452480. doi: 10.3389/fcimb.2024.1452480 (PMC11629706; doi:10.3389/fcimb.2024.1452480)
Supplement: Supplementary file 1 [file DataSheet1.zip › mendelian test/ebi-a-GCST90027650.csv_leave_one_out.pdf]

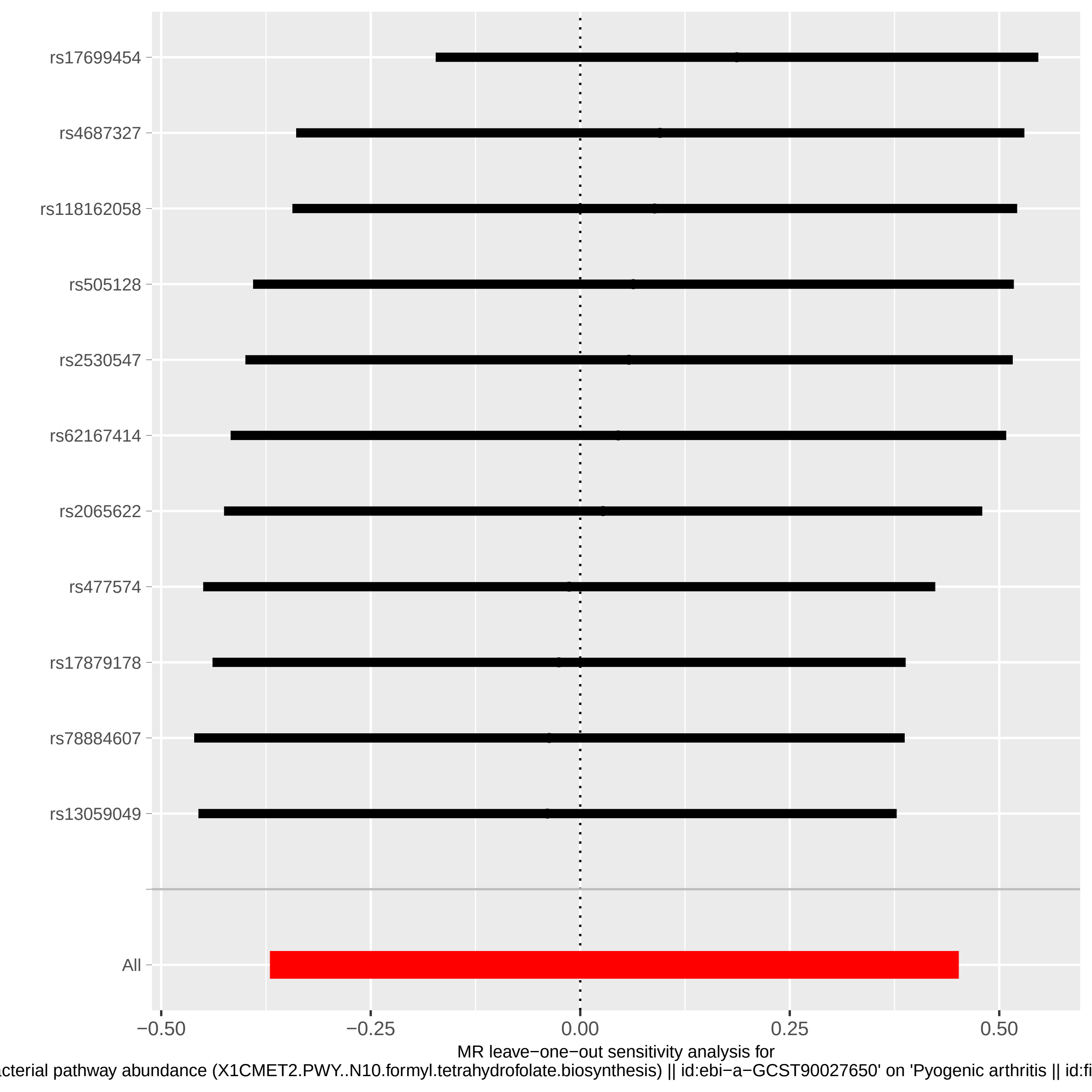
rs17699454
rs4687327
rs118162058
rs505128
rs2530547
rs62167414
rs2065622
rs477574
rs17879178
rs78884607
rs13059049
All
−0.50
−0.25
0.00
0.25
0.50
MR leave−one−out sensitivity analysis for
cterial pathway abundance (X1CMET2.PWY..N10.formyl.tetrahydrofolate.biosynthesis) || id:ebi−a−GCST90027650' on 'Pyogenic arthritis || id:fi
